# The Psychology of Existential Risk: Moral Judgments about Human Extinction

**DOI:** 10.1038/s41598-019-50145-9

**Published:** 2019-10-21

**Authors:** Stefan Schubert, Lucius Caviola, Nadira S. Faber

**Affiliations:** 1 Department of Experimental Psychology, University of Oxford, New Radcliffe House, Radcliffe Observatory Quarter, Woodstock Road, OX2 6GG Oxford, United Kingdom; 2 Oxford Uehiro Centre for Practical Ethics, University of Oxford, 16-17 St Ebbes St, Oxford, OX1 1PT United Kingdom; 3 College of Life and Environmental Sciences, University of Exeter, Washington Singer Building, Exeter, EX4 4QG United Kingdom

**Keywords:** Human behaviour, Psychology and behaviour

## Abstract

The 21st century will likely see growing risks of human extinction, but currently, relatively small resources are invested in reducing such existential risks. Using three samples (UK general public, US general public, and UK students; total N = 2,507), we study how laypeople reason about human extinction. We find that people think that human extinction needs to be prevented. Strikingly, however, they do not think that an extinction catastrophe would be uniquely bad relative to near-extinction catastrophes, which allow for recovery. More people find extinction uniquely bad when (a) asked to consider the extinction of an animal species rather than humans, (b) asked to consider a case where human extinction is associated with less direct harm, and (c) they are explicitly prompted to consider long-term consequences of the catastrophes. We conclude that an important reason why people do not find extinction uniquely bad is that they focus on the immediate death and suffering that the catastrophes cause for fellow humans, rather than on the long-term consequences. Finally, we find that (d) laypeople—in line with prominent philosophical arguments—think that the quality of the future is relevant: they do find extinction uniquely bad when this means forgoing a utopian future.

## Introduction

The ever-increasing powers of technology can be used for good and ill. In the 21^st^ century, technological advances will likely yield great benefits to humanity, but experts warn that they will also lead to growing risks of human extinction^1–4^. The risks stem both from existing technologies such as nuclear weapons, as well as emerging technologies such as synthetic biology and artificial intelligence^5^. A small but growing number of research institutes, such as the University of Oxford’s *Future of Humanity Institute* and the University of Cambridge’s *Centre for the Study of Existential Risk*, are studying these risks and how to mitigate them. Yet besides them, relatively small resources are explicitly devoted to reducing these risks.

Here, we study the general public’s views of the badness of human extinction. We hypothesize that most people judge human extinction to be bad. But *how bad* do they find it? And *why* do they find it bad? Besides being highly policy-relevant, these questions are central for humanity’s understanding of itself and its place in nature. Human extinction is a pervasive theme in myths and religious writings^6–10^.

One view is that human extinction is bad primarily because it would harm many concrete individuals: it would mean the death of all currently living people. On this view, human extinction is a very bad event, but it is not much worse than catastrophes that kill *nearly* all currently living people—since the difference in terms of numbers of deaths would be relatively small. Another view is that the human extinction is bad primarily because it would mean that the human species would go extinct and that humanity’s future would be lost forever. On this view, human extinction is *uniquely* bad: much worse even than catastrophes killing nearly everyone, since we could recover from them and re-build civilization. Whether extinction is uniquely bad or not depends on which of these considerations is the stronger: the immediate harm, or the long-term consequences.

Here is one way to pit these considerations against each other. Consider three outcomes: no catastrophe, a catastrophe killing 80% (near-extinction), and a catastrophe killing 100% (extinction). According to both considerations, no catastrophe is the best outcome, and extinction the worst outcome. But they come apart regarding the *relative differences* between the three outcomes. If the immediate harm is the more important consideration, then *the first difference*, between no catastrophe and near-extinction, is greater than *the second difference*, between near-extinction and extinction. That is because the first difference is greater in terms of numbers of harmed individuals. On the other hand, if the long-term consequences are more important, then the second difference is greater. The first difference compares two non-extinction outcomes, whereas the second difference compares a non-extinction outcome with an extinction outcome—and only the extinction outcome means that the future would be forever lost.

This thought-experiment was conceived by the well-known philosopher Derek Parfit^11^ (we have adapted the three outcomes slightly; see the Methods section). Parfit argued that most people would find the first difference to be greater, but he himself thought that the second difference is greater. Many other philosophers and other academics working to reduce the risk of human extinction agree with Parfit^12–15^. On their view, the badness of human extinction is greatly dependent on how long the future would otherwise be, and what the quality of future people’s lives would be. As the philosopher Nick Bostrom notes, predictions about the long-term future have often been left to theology and fiction, whilst being neglected by science^16^. However, in recent years, researchers have tried to assess what the long-term future may be like. They argue that if humanity does not go extinct, then the future could be both extraordinarily long and extraordinarily good, involving much greater quality of life than the current world. For instance, Nick Bostrom argues that a conservative estimate of humanity’s future potential is “at least 10^16^ human lives of normal duration”, which could “be considerably better than the average contemporary human life, which is so often marred by disease, poverty [and] injustice”^17^. He goes on to argue that less conservative estimates would yield even greater numbers, and a drastically improved quality of life. The argument is that if humanity develops to a sufficiently high technological level, then it will either cause its own extinction via misuse of powerful technologies, or use those technological powers to greatly improve the level of well-being. Furthermore, they argue, based on the view that new happy people coming into existence is morally valuable^11^, that it is of paramount moral importance to make sure that we realize our future potential, and prevent human extinction.

While philosophers have discussed the ethics of human extinction for some time, the general public’s views on this matter has not received much study. There are some studies on perceptions of risk of extinction, however. Two studies found that a slight majority do not think that humanity will go extinct, and that most of those who thought that it would go extinct thought that would happen at least 500 years into the future^18,19^. There is also a related literature on catastrophic risk in general, focusing primarily on non-extinction catastrophes. For instance, it has been argued that the fact that people use the availability heuristic—they focus on risks which have salient historical examples—leads to a neglect of new types of risks and risks of major catastrophes (which are rare, and therefore less psychologically available)^20^. Similarly, it has been argued that the fact that risk mitigation is a public good leads to under-investment, since it means that it is not possible to exclude free riders from benefiting from it^21^. On specific risks, there is a literature on the psychology of climate change showing that people fail to act to mitigate climate change because they engage in temporal discounting^22,23^ and motivated reasoning about its severity^24^, and because of psychological distance^25^ (e.g., temporal and social distance). However, to date there have been no studies on how laypeople reason about the moral aspect of human extinction: how bad it would be. Is the extinction of our own species something people care about? Do they recognize it as being fundamentally different in quality from other catastrophes? And if so, why?

## Results

### Study 1

In Study 1 (US sample, *n* = 183, mean age 38.2, 50.81% female), we studied the general public’s judgments of the badness of human extinction. A large majority of the participants (78.14%, 143/183 participants) found human extinction to be bad on a binary question (bad vs. not bad), and we got similar results on a seven-point scale (1 = *definitely not bad*, 4 = *midpoint*, 7 = *definitely bad*; *M* = 5.61; *SD* = 2.11). Participants also felt strongly that human extinction needs to be prevented (1 = *not at all*, 4 = *midpoint*, 7 = *very strongly*; *M* = 6.01, *SD* = 1.65), that they have a moral obligation to prevent it (1 = *definitely no*, 4 = *midpoint*, 7 = *definitely yes*; *M* = 5.69, *SD* = 1.86), and that funding work to reduce the risk of human extinction is more important than funding other areas of government, such as education, health care and social security (1 = *much less important to fund work to reduce the risk of human extinction*, 4 = *midpoint*, 7 = *much more important*; *M* = 5.43, *SD* = 1.72). Participants believed that provided that humanity will not go extinct, the future is going to be roughly as good as the present (1 = *much worse than the present world*, 4 = *about as good as the present world*, 7 = *much better than the present world*; *M* = 4.48, *SD* = 1.57), and the better they thought the future would be, the worse they considered extinction to be (*r* = 0.51, *P* < 0.001), as measured by the seven-point scale. Similarly, more optimistic^26^ participants judged extinction to be worse (*r* = 0.32, *P* < 0.001). Participants’ responses to the question whether the world gets better if a happy person comes into existence were close to the midpoint (1 = *definitely not better*, 4 = *midpoint*, 7 = *definitely better*; *M* = 4.45, *SD* = 1.73), and people who thought that that would make the world better were more likely (*r* = 0.22, *P* = 0.003) to find extinction bad. For further details about the results, see Supplementary Materials.

### Study 2a

**Figure 1:**
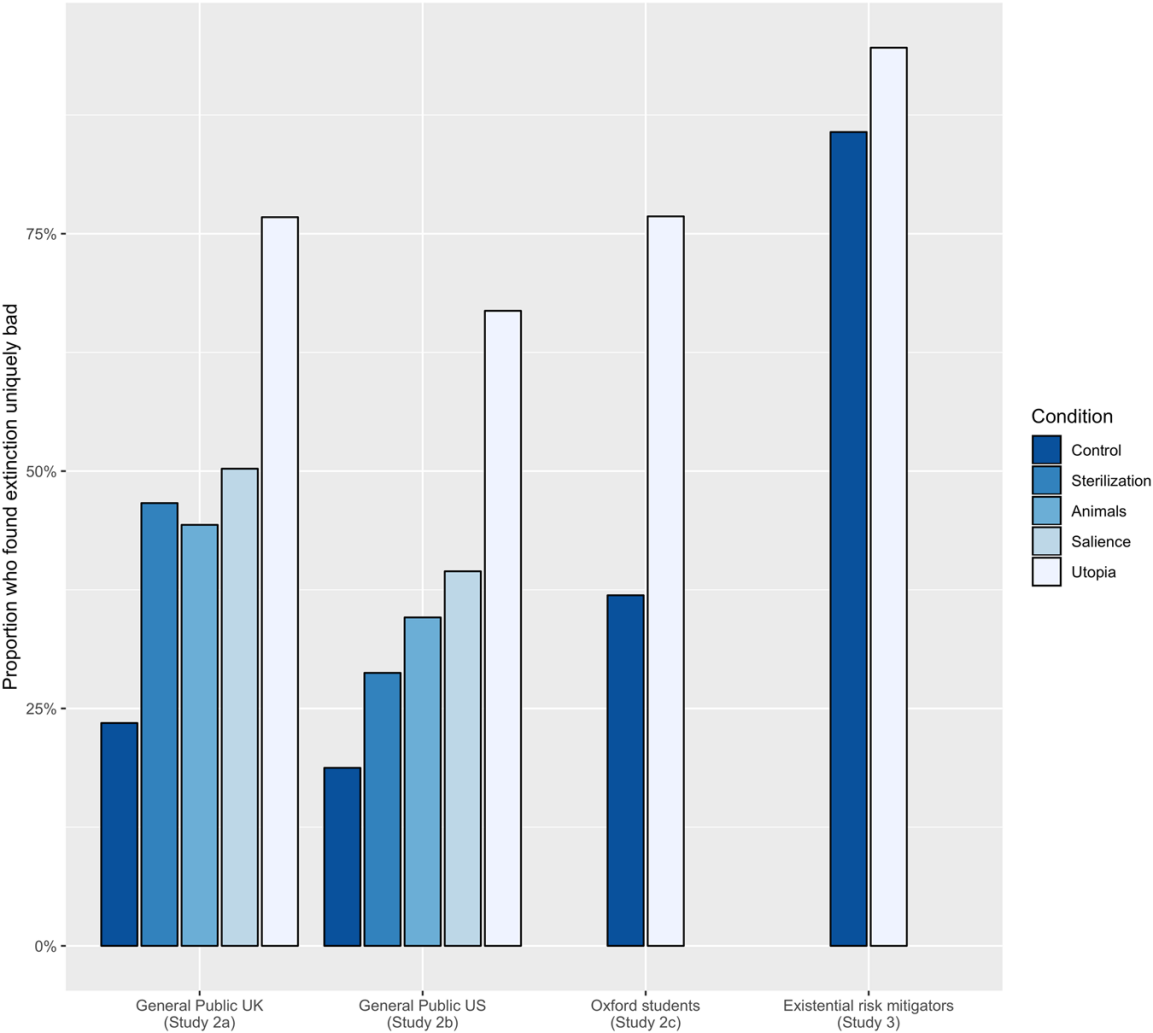
Proportions of participants who found extinction uniquely bad. (This means that they found the difference, in terms of badness, between a catastrophe killing 80% and a catastrophe killing 100% to be greater than the difference between no catastrophe and a catastrophe killing 80%.) Laypeople consistently did not find extinction uniquely bad in the control condition (*Control*), but did so in a scenario where the future would be very long and good conditional on survival (*Utopia*). The animals condition (*Animals*), sterilization condition (*Sterilization*) and salience condition (*Salience*) yielded in-between results. People explicitly devoted to existential risk reduction (*Existential risk mitigators*) consistently found extinction uniquely bad.

Having thus observed that people do find human extinction bad, we turned to studying whether they find it *uniquely* bad relative to non-extinction catastrophes in Study 2a (pre-registered at https://osf.io/hj2n4; British sample). Participants (*n* = 1,251, mean age 36.6, 35.33% female) were randomly divided into a control condition and four experimental conditions: “the animals condition”, “the “sterilization condition”, “the salience condition” and “the utopia condition” (see below for explanations of the manipulations). Participants in the control condition (257 participants) were presented with the three outcomes described above—no catastrophe, a catastrophe killing 80%, and a catastrophe killing 100%—and were asked how they would rank them from best to worst. As Parfit expected, a large majority (82.88%, 213/257 participants, cf. Fig. 1) ranked no catastrophe as the best outcome and 100% dying as the worst outcome. However, this was just a preliminary question: as per the discussion above, what we were primarily interested in was which difference participants that gave the expected ranking found greater: the first difference (meaning that extinction is not uniquely bad) or the second difference (meaning that extinction is uniquely bad). (Recall that the first difference was the difference between no catastrophe and a catastrophe killing 80%, and the second difference the difference between a catastrophe killing 80% and a catastrophe killing 100%.) We therefore asked participants who gave the expected ranking (but not the other participants) which difference they judged to be greater. We found that most people did not find extinction uniquely bad: only a relatively small minority (23.47%, 50/213 participants) judged the second difference to be greater than the first difference.

As stated, we included four experimental conditions aiming to explain these results. We thought that one reason why participants do not find extinction uniquely bad in the control condition is that they feel strongly for the victims of the catastrophes. Therefore, they focus on the immediate suffering and death that the catastrophes cause, which leads them to judge the difference between no one dying and 80% dying to be greater than the difference between 80% dying and 100% dying. To test this hypothesis, we included two conditions designed to trigger a weaker focus on the immediate harm. First, we included a condition where the catastrophes affected an animal species (zebras) rather than humans (“the animals condition”; otherwise identical to the control condition; 246 participants). (We chose zebras because zebra extinction would likely have small effects on humans; in contrast to extinction of, for example, pigs or dogs.) We hypothesized that people focus less on the immediate harm that the catastrophes cause if the catastrophes affect animals rather than humans^27^. Second, we included a condition where the catastrophes led to 80%/100% of the world’s population being unable to have children, rather than getting killed (“the sterilization condition”; otherwise identical to the control condition; 252 participants). We hypothesized that people would focus less strongly on the immediate harm that the catastrophes cause if they lead to sterilization rather than death.

Thus, we hypothesized that a greater share of the participants who gave the expected ranking would find extinction uniquely bad in the animals condition and the sterilization condition than in the control condition. We found, first, that a large majority ranked no catastrophe as the best outcome and 100% dying as the worst outcome (the expected ranking) both in the animals condition (89.84%, 221/246 participants) and the sterilization condition (82.54%, 208/252 participants). Subsequently, we found that our hypotheses were confirmed. The proportion of the participants who gave the expected ranking that found extinction uniquely bad was significantly larger (*χ*^2^(1) = 8.82, *P* = 0.003) in the animals condition (44.34%, 98/221 participants) than in the control condition (23.47%, 50/213 participants). Similarly, the proportion of the participants who gave the expected ranking that found extinction uniquely bad was significantly larger (*χ*^2^(1) = 23.83, *P* < 0.001) in the sterilization condition (46.63%, 97/208 participants) than in the control condition (23.47%, 50/213 participants).

We had another hypothesis for why control condition participants do not find extinction uniquely bad, namely that they neglect the long-term consequences of the catastrophes. To test this hypothesis, we included a condition where we made the long-term consequences salient (“the salience condition”; 248 participants). This condition was identical to the control condition, with the exception that we added a brief text explicitly asking the participants to consider the long-term consequences of the three outcomes. It said that if humanity does not go extinct (including if it suffers a non-extinction catastrophe, from which it can recover) it could go on to a long future, whereas that would not happen if humanity went extinct (see the Methods section for the full vignette). We also wanted to know whether participants see empirical information about the quality of the future as relevant for their judgments of the badness of extinction. Does it make a difference how good the future will be? We therefore included a maximally positive scenario, the “utopia condition” (248 participants), where it was said that provided that humanity does not go extinct, it “goes on to live for a very long time in a future which is better than today in every conceivable way”. It was also said that “there are no longer any wars, any crimes, or any people experiencing depression or sadness” and that “human suffering is massively reduced, and people are much happier than they are today” (in the scenario where 80% die in a catastrophe, it was said that this occurred after a recovery period; see the Methods section for the full text). Conversely, participants were told that if 100% are killed, then “no humans will ever live anymore, and all of human knowledge and culture will be lost forever.” We hypothesized that both of these manipulations would make more participants judge extinction to be uniquely bad compared with the control condition.

We found again that a large majority ranked no catastrophe as the best outcome and 100% dying as the worst outcome (the expected ranking) both in the salience condition (77.82%, 193/248 participants) and the utopia condition (86.69%, 215/248 participants). Subsequently, we found that our hypotheses were confirmed. The proportion of the participants who chose the expected ranking that found extinction uniquely bad was significantly larger (*χ*^2^(1) = 29.90, *P* < 0.001) in the salience condition (50.25%, 97/193 participants) than in the control condition (23.47%, 50/213 participants). Similarly, the proportion of the participants who chose the expected ranking that found extinction uniquely bad was significantly larger (*χ*^2^(1) = 30.30, *P* < 0.001) in the utopia condition (76.74%, 165/215 participants) than in the control condition (23.47%, 50/213 participants). We also found that there was a significant difference between the utopia condition and the salience condition (*χ*^2^(1) = 29.90, *P* < 0.001).

Our interpretation of these results is as follows. The utopia manipulation effectively does two things: it highlights the long-term consequences of the outcomes, and it says that unless humanity goes extinct, those consequences are going to be extraordinarily good. The salience manipulation only highlights the long-term consequences. Thus, we can infer that merely highlighting the long-term consequences make people more likely to find extinction uniquely bad, and that adding that the long-term future will be extraordinarily good make them still more likely to find extinction uniquely bad.

Lastly, we found that across all conditions, the more cognitively reflective the participants were (as measured by the Cognitive Reflection Test^28^), the more likely they were to judge extinction to be uniquely bad (*Exp*(*B*) = 0.15, *P* = 0.01, Odds ratio = 1.6).

In conclusion, we find that people do not find extinction uniquely bad when asked without further prompts, and have identified several reasons why that is. As evidenced by the animals and the sterilization conditions, they focus on the immediate harm that the catastrophes cause, because they feel strongly for the victims of the catastrophes—and on that criterion, near-extinction is almost as bad as extinction. As evidenced by the salience condition, they neglect the long-term consequences of the outcomes. We also find that participants’ empirical beliefs about the quality of the future make a difference: telling participants that the future will be extraordinarily good makes them significantly more likely to find extinction uniquely bad.

### Study 2b

To find out whether these results would hold up with different demographics, we aimed to replicate them using a sample of the US general public (pre-registered at https://osf.io/8amxs; *N* = 855, mean age 36.85, 48.65% female) in Study 2b. We found again that large majorities ranked no catastrophe as the best outcome and 100% dying as the worst outcome (the expected ranking) in the control condition (87.80%, 144/164 participants), the animals condition (92.44%, 159/172 participants), the sterilization condition (91.62%, 153/167 participants), the salience condition (83.05%, 147/177 participants) and the utopia condition (89.71%, 157/175 participants). And again we found that only a small minority of the participants who chose the expected ranking judged extinction to be uniquely bad in the control condition (18.75%, 27/144 participants). The proportion of the participants who chose the expected ranking who found extinction uniquely bad was significantly larger in the animals condition (34.59%, 55/159 participants; *χ*^2^(1) = 8.82, *P* = 0.003), the salience condition (39.45%, 58/147 participants; *χ*^2^(1) = 14.10, *P* < 0.001) and the utopia condition (66.88%, 105/157 participants; *χ*^2^(1) = 68.72, *P* < 0.001) than in the control condition. We also again found a significant difference between the utopia condition and the salience condition (*χ*^2^(1) = 21.868, *P* < 0.001). However, in the sterilization condition, only 28.75% (44/153 participants) of the participants who chose the expected ranking found extinction uniquely bad, which meant that the difference with the control condition was not significant on the 0.05-level (*χ*^2^(1) = 3.55, *P* = 0.059). Lastly, we found again that (across all conditions) the more cognitively reflective the participants were (as measured by the Cognitive Reflection Test), the more likely they were to judge extinction to be uniquely bad (*Exp*(*B*) = 0.21, *P* = 0.005, Odds ratio = 1.2).

### Study 2c

To further test the robustness of our findings across different demographics, we conducted Study 2c as another replication, this time using a sample of University of Oxford students (*N* = 196, mean age 24.27, 61% female). We only included the control and the utopia conditions. We found again that most participants ranked no catastrophe as the best outcome and 100% dying as the worst outcome (the expected ranking) both in the control condition (65.7%, 65/99 participants) and the utopia condition (84.5%, 82/97 participants). We then found again that a minority of the participants who chose the expected ranking found extinction to be uniquely bad in the control condition (36.92%, 24/65 participants), though this minority was slightly larger than in the two samples of the general public (cf. Fig. 1). We also found again that the proportion of the utopia condition participants who chose the expected ranking that found extinction uniquely bad (76.83%, 63/82 participants) was significantly larger (*χ*^2^(1) = 22.28, *P* < 0.001) than in the control condition. (These findings were further supported by five supplementary studies; see Supplementary Materials).

### Study 3

In Studies 2a to 2c, we thus found that when asked without further prompts, laypeople do not find extinction uniquely bad. In Study 3 (*N* = 71, mean age 30.52, 14.00% female) we aimed to test whether people devoted to preventing human extinction (existential risk mitigators) judge human extinction to be uniquely bad already when asked without further prompts. (Existential risks also include risks that threaten to drastically curtail humanity’s potential^12–15^, without causing it to go extinct, but we focus on risks of human extinction.) This would support the validity of our task by demonstrating a link between participants’ responses and behavior in the real world.

We recruited participants via the Effective Altruism Newsletter and social media groups dedicated to existential risk reduction, and only included respondents who put down reducing existential risk as their “most important cause”. Again we had two conditions, the control condition and the utopia condition. We hypothesized that a majority of participants would find extinction uniquely bad in both conditions. We found again that most participants ranked no catastrophe as the best outcome and 100% dying as the worst outcome (the expected ranking) both in the control condition (90.32%, 28/31 participants) and the utopia condition (92.50%, 37/40 participants). But unlike the samples in Studies 2a to 2c, and in line with our hypotheses, substantial majorities of the participants who chose the expected ranking found extinction uniquely bad both in the control condition (85.71%, 24/28 participants) and the utopia condition (94.59%, 35/37). The difference between the conditions was not significant (*χ*^2^(1) = 0.63, *P* = 0.43). In contrast to laypeople, existential risk mitigators thus found human extinction to be uniquely bad even when the description of the outcomes did not include information about the quality of the future. This suggests that judging human extinction to be uniquely bad, as measured by our task, may be a key motivator for devoting oneself to preventing it.

## Discussion

Our studies show that people find that human extinction is bad, and that it is important to prevent it. However, when presented with a scenario involving no catastrophe, a near-extinction catastrophe and an extinction catastrophe as possible outcomes, they do not see human extinction as *uniquely* bad compared with non-extinction. We find that this is partly because people feel strongly for the victims of the catastrophes, and therefore focus on the immediate consequences of the catastrophes. The immediate consequences of near-extinction are not that different from those of extinction, so this naturally leads them to find near-extinction almost as bad as extinction. Another reason is that they neglect the long-term consequences of the outcomes. Lastly, their empirical beliefs about the quality of the future make a difference: telling them that the future will be extraordinarily good makes more people find extinction uniquely bad.

Thus, when asked in the most straightforward and unqualified way, participants do not find human extinction uniquely bad. This could partly explain why we currently invest relatively small resources in reducing existential risk. However, these responses should not necessarily be seen as reflecting people’s well-considered views on the badness of human extinction. Rather, it seems that they partly reflect the fact that people often fail to consider the long-term consequences of extinction. Our studies suggest that it could be that if people reflected more carefully, they might to a greater extent agree that extinction is uniquely bad. A suggestive finding with regards to this is that higher scores on the cognitive reflection test predicted a greater tendency to find extinction uniquely bad. This could mean that deliberative thought-processes lead to finding extinction uniquely bad, whereas intuitive thought-processes lead to the opposite conclusion. More research is needed on the role of deliberation and intuition, as well as many other questions, such as the role of cognitive ability, and the ultimate evolutionary causes of why humans struggle to think clearly about their own extinction.

Finally, let us consider possible policy implications. If it is right that human extinction is uniquely bad, then we should arguably invest much more in making sure it does not happen. We should also change policy in many other ways; e.g., shift technology policy in a more cautious direction^29^. On this view, we should, if necessary, be prepared to make substantial sacrifices in order to make sure that humanity realizes its future potential. Hence much hinges on the complex question whether we deem our own extinction to be uniquely bad.

## Methods

All studies were approved by the University of Oxford’s Central University Research Ethics Committee (approval number: R56657/RE002) and participants in each study gave their informed consent beforehand. All studies were performed in accordance with relevant guidelines and regulations.

### Study 1

#### Participants

We recruited 210 participants and excluded 27 for not completing the study or failing the attention check.

#### Procedure

Level of optimism was measured by asking participants “how optimistic are you in general?” (where optimistic people were defined as “people who look to the future with confidence and who mostly expect good things to happen”)^26^. In addition to the measures reported above, we gave the Oxford Utilitarianism Scale, the Cognitive Reflection Test and demographic questions to the participants. The Oxford Utilitarianism Scale^30^ consists of two subscales: the impartial beneficence (IB) subscale and the instrumental harm (IH) subscale. The OUS-IB measures the degree to which someone values maximizing overall welfare, independent of its recipient. The OUS-IH measures the degree to which someone is willing to accept that harm is done in order to maximize overall welfare. The Cognitive Reflection Test^28^ measures the tendency to answer questions reflectively and resist reporting the first response that comes to mind.

### Study 2a

#### Participants

We recruited 1301 participants via Prolific and excluded 50 for not completing the study or failing an attention check. The study was pre-registered at https://osf.io/hj2n4/.

#### Procedure

Participants were first asked to consider three outcomes, A, B and C, and rank them from best to worst. The outcomes in the control condition were described as follows:

**The control condition:**

(A) There is no catastrophe.
(B) There is a catastrophe that immediately kills 80% of the world’s population.
(C) There is a catastrophe that immediately kills 100% of the world’s population.

This meant that our text differed from Parfit’s^13^ as follows. We used “no catastrophe” rather than Parfit’s “peace” because we thought that “peace” had positive associations that could be a potential confounder. We used “a catastrophe” rather than Parfit’s “a nuclear war” because we thought that there was no reason to specify the nature of the catastrophe. And we said that 80%, rather than 99%, die in the non-extinction catastrophe, to make it more plausible that humanity could recover.

The outcomes in the other conditions were described as follows:

**The animals condition:**

(A) There is no catastrophe.
(B) There is a catastrophe that immediately kills 80% of the world’s zebra population.
(C) There is a catastrophe that immediately kills 100% of the world’s zebra population.

**The sterilization condition:**

(A) There is no catastrophe.
(B) There is a catastrophe that immediately causes 80% of the world’s population to go sterile, meaning they cannot have children.
(C) There is a catastrophe that immediately causes 100% of the world’s population to go sterile, meaning they cannot have children.

**The salience condition:**

(A) There is no catastrophe.
(B) There is a catastrophe that immediately kills 80% of the world’s population.
(C) There is a catastrophe that immediately kills 100% of the world’s population.

Please rank these three outcomes from best to worst.

When you do so, please remember to consider **the long-term consequences** each scenario will have for humanity. If humanity does not go extinct, it could go on to a long future. This is true even if many (but not all) humans die in a catastrophe, since that leaves open the possibility of recovery. However, if humanity goes extinct (if 100% are killed), there will be no future for humanity.

**The utopia condition:**

(A) There is no catastrophe and humanity goes on to live for a very long time in a future which is better than today in every conceivable way. There are no longer any wars, any crimes, or any people experiencing depression or sadness. Human suffering is massively reduced, and people are much happier than they are today.
(B) There is a catastrophe that immediately kills 80% of the world’s population. However, humanity eventually recovers to its original size, and then goes on to live for a very long time in a future which is better than today in every conceivable way. There are no longer any wars, any crimes, or any people experiencing depression or sadness. Human suffering is massively reduced, and people are much happier than they are today.
(C) There is a catastrophe that immediately kills 100% of the world’s population. This means that humanity will go extinct, that no humans will ever live anymore, and all of human knowledge and culture will be lost forever.

On the next page, participants who ranked A as the best outcome and C as the worst were again presented with the three outcomes (other participants were excluded), and told:

“We are now interested in your views of how much better A is than B, and how much better B is than C. In terms of badness, which difference is greater: the difference between A and B, or the difference between B and C?”

In addition to the measures reported above, we gave the Oxford Utilitarianism Scale and demographic questions to participants (see Supplementary Materials for results).

### Study 2b

#### Participants

We recruited 994 participants and excluded 139 for not completing the study or failing an attention check. In addition to the measures reported above, we gave the Oxford Utilitarianism Scale and demographic questions to the participants (see Supplementary Materials for results). The study was pre-registered at https://osf.io/8amxs.

#### Procedure

The procedure was the same as in study 2a.

### Study 2c

#### Participants

We recruited 204 participants and excluded 8 for not giving an answer. The procedure was the same as for study 2a, except only the control condition and the utopia condition were included. In addition to the measures reported above, we gave the Oxford Utilitarianism Scale and demographic questions to the participants (see Supplementary Materials for results).

### Study 3

#### Participants

We recruited 196 participants. However, since we were only interested in those effective altruists who consider existential risk mitigation to be the top cause area, only 83 were included into the analysis. 12 participants were excluded for failing an attention check. The final sample was 71 participants.

#### Procedure

The procedure was the same as for study 2a, except only the control condition and the utopia condition were included. In addition, participants were asked three questions assessing how uniquely bad they found extinction (extinction prevention questions). The first question concerned which scenario (A = a catastrophe kills 50% of the world’s population, but humanity recovers to its original size and goes on to live for a very long time, B = painless extinction) they would want to prevent (1 = *definitely A*, 4 = *midpoint*, 7 = *definitely B*). The second question asked if participants would rather support political party A, which works to reduce the risk of scenario A, or political party B, which works to reduce the risk of scenario B (1 = *definitely A*, 4 = *midpoint*, 7 = *definitely B*). The third question asked what they thought the morally right choice for government leaders would be if they had to choose between reducing the risk for either scenario A or B (1 = *definitely reduce the risk of A*, 4 = *midpoint*, 7 = *definitely reduce the risk of B*).

We also gave the Oxford Utilitarianism Scale, The Cognitive Reflection Test and demographic questions to participants. (See Supplementary Materials for additional results).

## Supplementary information

Supplementary Materials

## Data Availability

Reports of all measures, manipulations, and exclusions, and all data, analysis code, and experimental materials for all studies are available for download at: https://osf.io/pd9ca/.
