# Supplementary material for: The Psychology of Existential Risk: Moral Judgments about Human Extinction
Source: Sci Rep. 2019 Oct 21;9:15100. doi: 10.1038/s41598-019-50145-9 (PMC6803761; doi:10.1038/s41598-019-50145-9)
Supplement: Supplementary file 1 — Supplementary Materials [file 41598_2019_50145_MOESM1_ESM.pdf]

Supplementary Materials for

How bad is human extinction? The psychology of existential risk

Stefan Schubert, Lucius Caviola, Nadira S. Faber

Correspondence to: stefan.schubert@psy.ox.ac.uk

This PDF file includes:

Links to the Full Studies, Material, and Pre-Registrations
Supplementary results for study 1
Table S1
Supplementary results for study 2a
Table S2
Supplementary results for study 2b
Table S3
Supplementary results for study 2c
Table S4
Supplementary results for study 3
Table S5
Supplementary results for study S1
Supplementary results for study S2
Supplementary results for study S3
Supplementary results for study S4
Supplementary results for study S5

Links to the Full Studies, Material, and Pre-Registrations:
https://osf.io/pd9ca/

Supplementary results for study 1 - beliefs about extinction

Most participants found it unlikely that humanity will go extinct within the next 100 years (1 = *very unlikely*, 7 = *very likely*, $M = 2.51$, $SD = 1.76$), and the more likely participants found extinction to be, the less bad they found extinction to be ($r = -0.21$, $P = 0.004$). Finding extinction bad correlated with other variables as shown in Table S1. In addition, a *t*-test with

gender revealed that there was no significant difference between how much men and women felt extinction needed to be prevented.

Table S1. Correlations.

	**1**	**2**	**3**	**4**	**5**
1. Finding extinction bad	-				
2. Importance of preventing extinction	0.75***				
3. Personal moral obligation to prevent extinction	0.66***	0.78***			
4. Prioritize preventing extinction over other causes	0.49***	0.61***	0.61***		
5. Quality of the future	0.51***	0.44***	0.42***	0.30***	
6. Probability of extinction	-0.21**	-0.17*	-0.13	-0.12	-0.11
7. Happy person into existence good	0.22**	0.25***	0.22**	0.28***	0.18*
8. Optimism - pessimism	0.32***	0.30***	0.31***	0.23**	0.30***
9. OUS - impartial beneficence subscale	0.09	0.04	0.15*	0.10	0.05
10. OUS - instrumental harm subscale	0.05	-0.04	-0.05	-0.02	0.10
11. Cognitive Reflection Test	-0.03	0.06	0.13	0.13	-0.05
12. Gender	-0.09	-0.06	-0.09	-0.13	-0.11
13. Education	0.05	0.06	0.08	0.02	0.04
14. Income	0.05	0.02	0.11	-0.02	0.04
15. Age	0.14	0.12	0.15	0.03	0.05

16. Religiosity	0.20**	0.12	0.15*	0.03	0.07
17. Economic conservatism	0.14	0.06	0.08	0.02	0.19*
18. Social conservatism	0.17*	0.07	0.07	0.05	0.18*

p-values are marked as follows: * = significant at the .05 level, ** = significant at the .01 level, *** = significant at the .001 level.

Supplementary results for study 2a - UK sample

Finding the difference between outcomes B and C to be greater than the difference between outcomes A and B (finding extinction uniquely bad) correlated with other variables as follows (Table S2).

Table S2. Logistic regression with finding extinction uniquely bad as the predicted variable.

**Measure**	**Odds ratio**	***p*-value**
OUS - instrumental harm subscale	1.04	.46
OUS - impartial beneficence subscale	0.94	.32
Cognitive Reflection Test (CRT)	1.16	.01*
Gender (male = 0, female = 1)	0.91	.63
Age	0.99	.13
Education	0.93	.30
Income	1.07	.17

p-values are marked as follows: * = significant at the .05 level, ** = significant at the .01 level, *** = significant at the .001 level. CRT and OUS were analyzed in one logistic regression, demographics in another logistic regression.

The utopia condition was called “the good future condition” in the pre-registration.

Supplementary results for study 2b - US sample (Mturk)

Finding the difference between outcomes B and C to be greater than the difference between outcomes A and B (finding extinction uniquely bad) correlated with other variables as follows (Table S3).

Table S3. Logistic regression with finding extinction uniquely bad as the predicted variable.

**Measure**	**Odds ratio**	***p*-value**
OUS - instrumental harm subscale	0.84	.008**
OUS - impartial beneficence subscale	1.00	1.000
Cognitive Reflection Test (CRT)	1.23	.005**
Gender (male = 0, female = 1)	0.66	.005**
Age	1.01	.202
Education	1.04	.569
Income	0.98	.593

p-values are marked as follows: * = significant at the .05 level, ** = significant at the .01 level, *** = significant at the .001 level.

Again, the utopia condition was called "the good future condition" in the pre-registration.

Supplementary results for study 2c - Oxford students

Finding the difference between outcomes B and C to be greater than the difference between outcomes A and B (finding extinction uniquely bad) correlated with other variables as follows (Table S4).

Table S4. Logistic regressions with finding extinction uniquely bad as the predicted variable.

Measure	Odds ratio	*p*-value
OUS - instrumental harm subscale	1.29	.052
OUS - impartial beneficence	0.85	.250
Gender (male = 0, female = 1)	0.47	.036*
Age	0.98	.413

p-values are marked as follows: * = significant at the .05 level, ** = significant at the .01 level, *** = significant at the .001 level. Consistent with our other studies, OUS and demographics were analyzed in two separate logistic regressions.

Supplementary materials for study 3 - Effective Altruists

As reported in the paper, almost all participants found the difference between extinction and a catastrophe to be more relevant than the difference between a catastrophe and no catastrophe. We therefore did not conduct logistic regressions with this variable and instead conducted correlational analyses with the mean of the three extinction prevention questions across the control condition and the utopia condition (Table S5). In addition, a *t*-test with gender revealed that there was a significant difference between how much men (M = 5.84, SD = 1.46) and women (M = 4.64, SD = 1.79) felt extinction needed to be prevented, $t(14.15) = 2.17$, $P = .04$, $d = 0.79$. Overall, participants strongly favored preventing extinction both in the control condition (M = 5.54, SD = 1.55) and the utopia condition (M = 5.74, SD = 1.60).

Table S5. Correlations with the mean of the three extinction prevention questions.

Measure	Coefficient	*p*-value
OUS - instrumental harm subscale	0.32	.007**

OUS - impartial beneficence	0.03	.793
CRT	-0.03	.822
Age	-0.26	.028*

p-values are marked as follows: * = significant at the .05 level, ** = significant at the .01 level, *** = significant at the .001 level.

Supplementary studies

We conducted five supplementary studies (studies S1 to S5; total N=1625). Their results are broadly in line with the findings reported in the main text.

Study S1

Study S1 (pre-registered) was identical to studies 2a and 2b reported in the main text, with the exception that we only had two conditions in study S1: the control condition and the utopia condition. The results were in line with the effects of the utopia manipulation in studies 2a, 2b and 2c. Out of our final sample of 182 participants, large majorities ranked no catastrophe as the best outcome and 100% dying as the worst outcome both in the control condition (92.40%, 85/92) and the utopia condition (90.00%, 81/90). Subsequently, we found, in line with our pre-registered hypothesis, that the proportion of participants who found the difference between 80% dying and 100% dying the largest was significantly larger ($\chi^2(1) = 29.439$, $P < .001$) in the utopia condition (69.14%, 56/81 participants) than in the control condition (25.88%, 22/85). Hence, the results are convergent with those of studies 2a, 2b, and 2c that are reported in the main text.

Study S2

Studies S2-S4 (all pre-registered) employed another paradigm. Here, participants were asked to compare painless extinction with a catastrophe killing 50% of all humans (after which point "humanity recovers to its original size in a few centuries, and then goes on to live for a very long time").

In Study 2 (final N=230), a majority (142 out of 230; 61.7%) of participants rated painless extinction as worse than a catastrophe killing 50%. We also asked three questions measuring on a seven point-scale which of the two scenarios would be more important to prevent. Using a merged variable over these three questions we found that participants valued preventing extinction significantly more than preventing the non-extinction catastrophe (M = 4.52, SD = 1.97), $t(229) = 3.99$, $P < .001$. These results contrasted with our pre-registered hypotheses: at the outset of our studies, we expected participants to find human extinction less bad than they actually do. Study 1 confirmed the finding of study S2, that people find human extinction very bad—however, as subsequent studies (studies 2a-2c) showed, they do not always view it as *uniquely bad* in comparison to non-extinction catastrophes.

Study S3

In study S3 (final N=427), we used the same dependent variables as in study S2, but here we had three conditions: one where the long-term future was said to be good, one where it was said to be bad, and one with no such information (control; same as in study S2). An ANOVA indicated that the merged variable from Study S2 varied significantly across conditions, $F(2, 424) = 29.00$, $P < .001$. In line with our pre-registered hypothesis, a *post hoc* Tukey test showed that each condition differed significantly from the others, with participants in the utopia condition (M = 5.01, SD = 1.74) valuing preventing extinction more (relative to preventing the non-extinction

catastrophe) than control condition participants (*M* = 4.46, *SD* = 2.11, *P* = .049), who in turn valued preventing extinction more than participants in the bad future condition (*M* = 3.31, *SD* = 1.95, *P* < .001). This finding further supports the results from studies 2a-2c reported in the main text, which show that people find extinction worse relative to non-extinction catastrophes, the better the future is predicted to be (conditional on non-extinction).

Study S4

In study S4 (final N=288), we again used the same dependant variables as in study S2, but added a condition where the catastrophe/extinction affected animals (zebras) rather than humans. In line with our pre-registered hypothesis, an independent-samples *t*-test on the merged variable (cf. study S2) showed that participants valued preventing the extinction of animals more highly (relative to preventing a catastrophe killing 50% of the animal population; *M* = 5.24, *SD* = 1.76) than they valued preventing human extinction (relative to preventing a catastrophe killing 50% of all humans; *M* = 4.74, *SD* = 1.89), *t*(286) = 2.31, *P* = .022. Hence, the results of study S4 are in line with the finding from studies 2a-2c reported in the main text, that participants find extinction worse (relative to non-extinction catastrophes) when the extinction/catastrophes affect animals rather than humans.

Study S5

Study S5 (pre-registered) was an initial attempt to study whether participants deem human extinction uniquely bad relative to non-extinction catastrophes. We used a paradigm that we in hindsight recognized as overly complex and potentially confusing to participants, and therefore not well-suited to test our research questions. Rather than directly and explicitly asking

participants about their judgments of the *relative* difference between extinction and non-extinction catastrophes (as we did in our later studies studies), in study S5 we asked participants (final N=398) to rate the badness of different catastrophes (resulting in the death of 25%, 50%, 75% or 100% of the population) and a non-catastrophe (killing no one) in *absolute* terms, with the aim to indirectly infer the relative differences from the absolute values later. We later realized that this method is not reliable, because participants may not realize what claims about the relative differences their judgments of the badness of the different outcomes commit them to. For that reason, we rather have to ask participants directly about the relative differences, as we did in studies 2a-2c and 3. Our confidence in the data yielded with this paradigm further decreased due to an overly high participant drop-out rate of nearly 35% (206 out of 604).

In one condition of study S5, we asked participants to focus on the short-term future, and in another to focus on the long-term future (cf. the salience condition from studies 2a-2c in the main text). We also had a control condition where we did not give any such instruction (cf. the control condition from the studies in the main text). We found that in the control condition participants did not deem extinction uniquely bad. That is, the participants indicated that the difference in badness between the catastrophes killing 75% and 100% was not greater than the difference in badness between the catastrophes killing 50% and 75% (in line with our pre-registered hypothesis and the findings reported in the main text). We also found that none of the two interventions introducing a certain focus on the future (short-term or long-term) changed these results (in contrast to our pre-registered hypothesis and the findings on from the “salience condition” reported in the main text). We refrain from interpreting these mixed results because of our doubts about the paradigm that we used.
